# Ultrasonographic Measurement of Torsional Side Difference in Proximal Humerus Fractures and Humeral Shaft Fractures: Theoretical Background with Technical Notes for Clinical Implementation

**DOI:** 10.3390/diagnostics12123110

**Published:** 2022-12-09

**Authors:** Jan-Niklas Menzel, Dafang Zhang, Christian Krettek, Nael Hawi, Sam Razaeian

**Affiliations:** 1 Hannover Medical School, Carl-Neuberg-Straße 1, 30625 Hannover, Germany; 2 Department of Orthopaedic Surgery, Brigham and Women’s Hospital, 75 Francis St, Boston, MA 02115, USA; 3 Hannover Humerus Registry (HHR), Traumastiftung gGmbH, Carl-Neuberg-Str. 1, 30625 Hannover, Germany; 4 Orthopaedic and Surgical Clinic Braunschweig (OCP), Mauernstraße 35, 38100 Braunschweig, Germany; 5 Department of Trauma Surgery, Hannover Medical School, Carl-Neuberg-Straße 1, 30625 Hannover, Germany

**Keywords:** proximal humerus fracture, humeral shaft fracture, humeral torsion, torsional side difference, ultrasonographic

## Abstract

Both nonoperative and operative treatment of proximal humerus fractures (PHF) and humeral shaft fractures can result in torsional side differences. Several measurement methods are available to determine torsional malalignment. While conventional X-ray or computed tomography would entail additional radiation exposure for the patient, and while magnetic resonance imaging might be associated with higher costs and is not suitable in cases of surgically treated fractures due to metal-induced artifacts, the sonographic measurement of humeral torsion represents a readily available and quickly performable measurement method without radiation exposure. Both fully sonographic procedures and sonographically assisted procedures have been described in the literature for this purpose. To date, however, its application in the case of trauma patients, for example those with healed PHF and humeral shaft fractures, is not reported. This viewpoint article aims to provide a concise summary of the literature concerning ultrasonographic indirect measurements of humeral torsional side differences, with technical notes for clinical implementation in case of healed proximal humerus fractures and humeral shaft fractures.

## 1. Introduction

Both nonoperative and operative treatments of proximal humerus fractures (PHF) and humeral shaft fractures can result in torsional side differences. Several anatomical, radiological, and sonographic measurement methods are known to determine torsional malalignment [1,2,3,4,5]. While the determination of humeral torsion with the aid of conventional X-ray diagnostics or computed tomography would entail additional radiation exposure for the patient, and while magnetic resonance imaging might be associated with higher costs and is not suitable in cases of surgically treated fractures due to metal-induced artifacts, the sonographic measurement of humeral torsion represents a readily available and quickly performable measurement method without radiation exposure. Both fully sonographic procedures and sonographically assisted procedures have been described in the literature for this purpose [4,6]. To date, however, its application in cases of trauma patients, for example those with healed PHF and humeral shaft fractures, is not reported. In 1987, Harland described a sonographic method for measuring humeral torsion in which the torsion angle could be read directly from the ultrasound device screen [4,7]. This later evolved into a sonographically assisted procedure, which was described in 1995 by Ito et al. In the modified technique, the torsion angle is not read directly from the screen of the device but is determined with the aid of an additionally used protractor either in relation to a horizontal [8,9,10] or a vertical [6,11,12,13,14,15,16,17] reference axis. This viewpoint article aims to provide a concise review of the literature concerning ultrasonographic indirect measurements of humeral torsional side differences with technical notes for clinical implementation in cases of healed proximal humerus fractures and humeral shaft fractures.

## 2. Materials and Methods

### 2.1. Clinical Approach and Setup

Ultrasonographic-assisted measurement of humeral torsion is suitable in cases of radiographically healed nonsurgically and surgically treated PHF, as well as humeral shaft fractures with clinical conspicuous side differences in range of rotational shoulder motion. This method might unmask the actual amount of osseous torsional deformity compared with the uninjured side. Bilateral fractures; fractures of the collum anatomicum; fractures with involvement of the bicipital groove, or loss of integrity between the groove and humeral head; as well as radiographic signs of delayed union or nonunion; and concomitant forearm fractures might be contraindications. The same applies to severe obesity and in cases of implanted prothesis.

**Figure 1:**
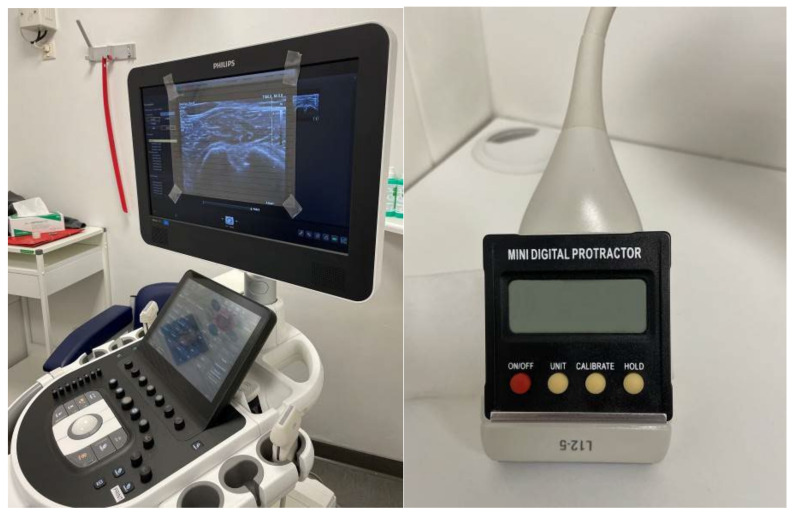
Equipment for sonographically assisted measurement of humeral torsion.

Ultrasonographic measurement is performed using a 7.5-MHz linear transducer of a conventional ultrasound scanner in B-mode (Affinity 70G, Koninklijke Philips N.V., 1096 BC Amsterdam, The Netherlands). The ultrasound scanner must be previously prepared with minor modifications by the examiner. A transparent film with horizontal parallel lines is attached to the screen of the ultrasound device and a conventional digital protractor (Junerain mini digital protractor, Shenzhen Si Hai Xin Zhou Technology Co Ltd., Danzhutou Comm. Nanwan Street, 518114 Shenzhen, China) is fixed to the transducer (Figure 1).

This indirect measurement of humeral torsion is performed with the patient lying supine. The patient’s upper extremity is positioned in 90° of shoulder abduction, 90° of elbow flexion, and 90° degrees of forearm supination. In addition, a cuff with an integrated metal plate is attached to the ulnar side of the patient’s distal forearm, which allows a second protractor to be magnetically attached to the distal forearm. Both protractors were zeroed on the horizontal table plane before attachment. 

**Figure 2:**
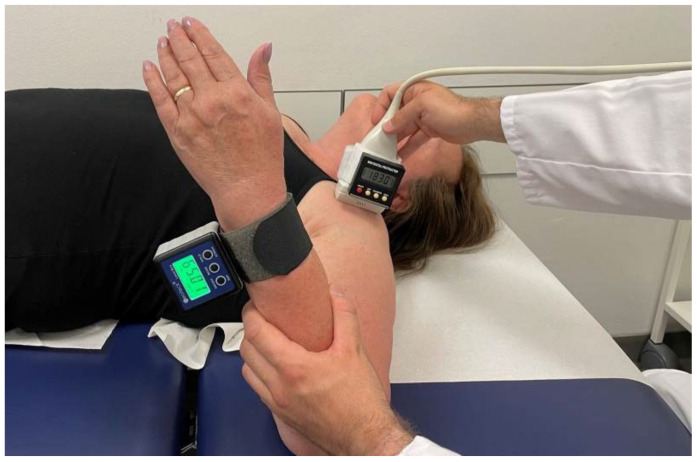
Sonographically assisted indirect measurement of humeral torsion. The angles determined are each given a positive sign if the corresponding protractor is inclined inward/caudal during the measurement and a negative sign if the protractor is inclined outward/cranial during the measurement. As the corresponding protractor attached to the transducer is inclined outward/cranial in this case, a negative sign must be given to this determined angle. As also the vertically positioned forearm protractor was zeroed on the horizontal table plane before attachment, its measured angle has to be subtracted from 90° to obtain the actual forearm angle. In this case, the forearm protractor displayed 65.01°, which results in 24.99° as actual forearm angle (90°–65.01°). All values were rounded for further calculation.

**Figure 3:**
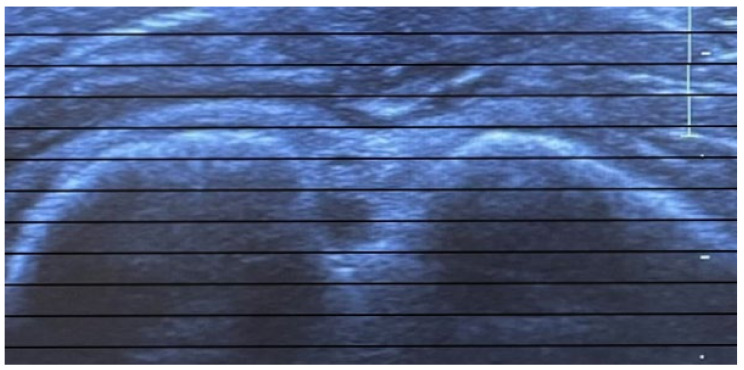
Greater and lesser tuberosities are displayed horizontally in one plane with the aid of a film with horizontal parallel lines attached to the screen of the ultrasound device.

At the beginning of the measurement, the ultrasound probe is placed on the shoulder and aligned perpendicular to the long axis of the humerus so that the intertubercular sulcus can be seen on the screen of the ultrasound device. Internal and external rotation of both the transducer and the forearm is then performed until the greater and lesser tuberosities are displayed horizontally in one plane on the ultrasound machine screen with the aid of the horizontal parallel lines of the attached film (Figure 2 and Figure 3).

In this position, the angles of the two protractors used are determined in relation to a vertical reference axis and noted to calculate humeral torsion. The angles determined are each given a positive sign if the corresponding protractor is inclined inward/caudal during the measurement and a negative sign if the protractor is inclined outward/cranial during the measurement (Figure 2). As also the vertically positioned forearm protractor was zeroed on the horizontal table plane before attachment, its measured angle has to be subtracted from 90° to obtain the actual forearm angle (Figure 2). 

**Figure 4:**
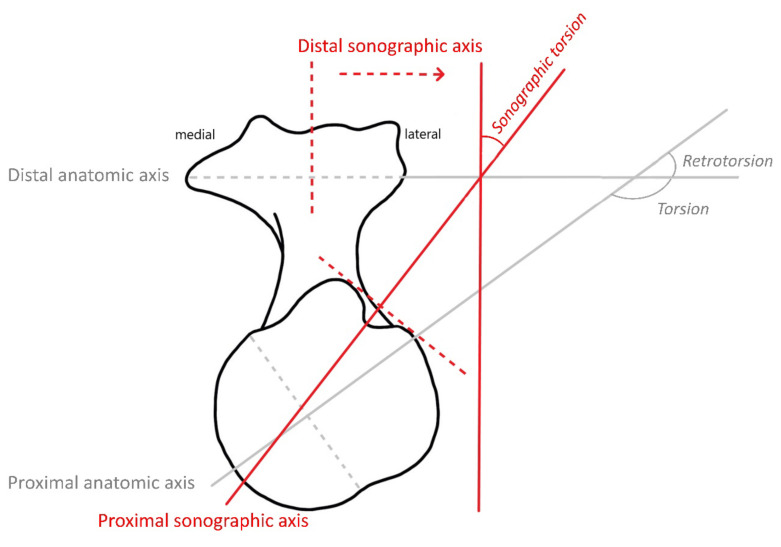
Humeral torsion, retrotorsion, and sonographic torsion in a right humerus.

Humeral torsion is determined indirectly with this method in relation to a vertical reference axis (Figure 4) and is calculated from the two determined, rounded angles according to the following formula:

Calculation of sonographic humeral torsion:

Sonographic humeral torsion=angle at forearm−angle at transducer

Example calculation for Figure 2:

$$Sonographic\;humeral\;torsion=25{}^{\circ}\;\left(angle\;at\;forearm\;\right)--18{}^{\circ}\;\left(angle\;at\;transducer\right)=43{}^{\circ}$$

### 2.2. Example Case Presentation

**Figure 5:**
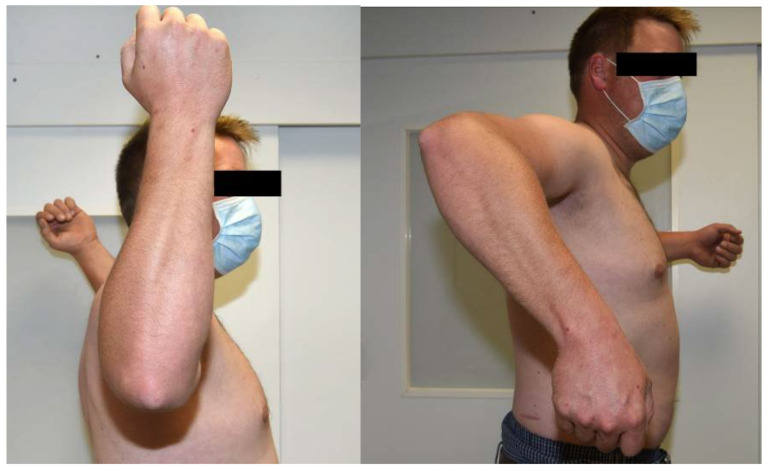
Case study: Active range of rotational motion of the unaffected shoulder.

**Figure 6:**
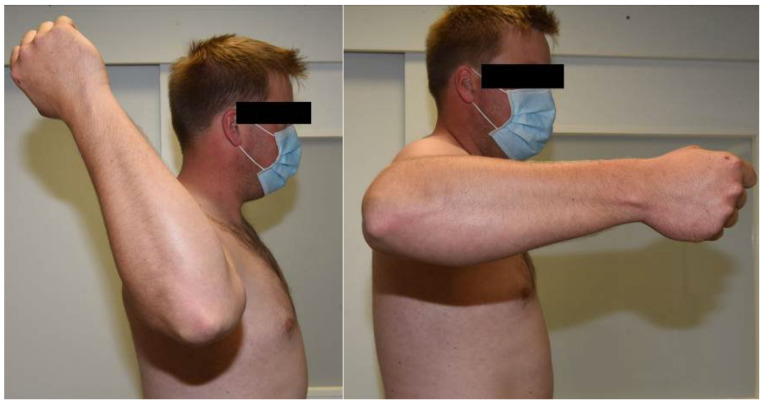
Case study: Active range of rotational motion of the affected shoulder.

A 29-year-old patient presented for clinical and radiological follow-up approximately three years after a surgically treated combined humeral shaft fracture and supracondylar humerus fracture. The patient developed paresis of the radial nerve during the healing process, which required revision surgery with neurolysis of the radial nerve. At the time of clinical radiological follow-up, the paresis of the radial nerve was completely recovered. However, a marked side difference in rotational range of motion of the shoulders was observed in the examination. Compared with the unaffected contralateral side, the formerly fractured right arm had decreased passive shoulder internal rotation and increased passive shoulder external rotation (Figure 5 and Figure 6 and Table 1). This side difference in shoulder rotational ability was perceived as very disturbing by the patient. Subsequent sonographically assisted measurement revealed a sonographic humeral torsion of −35° on the affected right side and 28° on the healthy left side, matching the suspicious clinically observed rotational deficits of the affected side. Based on these examination results, it can be concluded that the patient has a clinically relevant sonographic humeral torsional side difference of 63°.

## 3. Interpretation of Ultrasonographic Measurements

Humeral torsion is a measure of the physiological torsion of the humerus in its longitudinal axis and should not be confused with rotation in the shoulder joint. It is described by the torsion angle (Figure 4) and can be directly or indirectly determined using different anatomical, radiological, and sonographic measurement techniques [1,2,3,4,5]. The torsion angle results from the angular relationship of a defined axis at the proximal end of the humerus to another axis at the distal humerus (Figure 4). While many mammals have a torsion angle of approximately 90° due to a posteriorly directed proximal articular surface, humans are thought to have undergone inward rotation of the proximal articular surface during evolution, which can result in much higher torsion angles of 160° or more in humans [18,19]. Instead of humeral torsion, humeral retroversion or retrotorsion is often determined in the literature, which is the complementary angle to humeral torsion; furthermore, the sum of the humeral torsion and humeral retrotorsion equals 180° (Figure 4) [20]. On the contrary, the sonographically determined humeral torsion differs from the above mentioned anatomic humeral torsion in particular due to a different proximal axis. In addition, its values are reduced by about 90° due to the distal axis being vertical instead of horizontal (Figure 4). 

Physiological humeral torsion is different in each individual and can vary greatly depending on ethnicity or even the age of the individual [21]. Moreover, there remains disagreement in the literature whether, in addition to this high interindividual variability of humeral torsion, there are also intraindividual side differences in humeral torsion. For example, some authors have observed a lower humeral torsion in the dominant arm [22,23,24,25,26,27], whereas other authors have not found a significant side difference [6,11,28,29,30,31,32,33].

## 4. Discussion

Since the measurement methods for determining humeral torsion are based on the angular relationship of a defined proximal axis to another distal axis, the correct definition of these two axes is critical for the quality of the measurement results. The determination of the proximal joint axis proves to be particularly problematic, since clear landmarks for the determination of this axis are lacking in the region of the proximal humerus [34]. For the determination of the proximal joint axis [3,28,35,36] and the distal joint axis [3,28], different points of orientation are sometimes chosen in the literature, which is why the position of the two axes may differ slightly in various publications. As a result, torsion angles using different measurement methods, and sometimes even using the same measurement methods, can have very different values and cannot be directly compared with each other (Table 2). 

In the sonographic measurement procedures for determining humeral torsion, the proximal joint axis is usually determined assuming a constant angular relationship using the sulcus intertubercularis as a landmark [4,6,8,9,10,11,12,13,17,34,37]. However, in part due to a differing method of determining the distal joint axis, the torsion values of the sonographic measurement methods cannot always be directly compared with each other. 

The advantage of sonographic measurement methods are their ready availability and fast as well as simple performance. Moreover, patients are not exposed to any additional radiation. A disadvantage of this method lies in the fact that it is not a direct measurement of humeral torsion but rather allows indirect measurement based on an assumed constant angular relationship between the proximal joint axis and the sulcus intertubercularis. 

Advancing digitalization and the increasing availability of handheld ultrasound equipment has led to a rising use of sonographic examination techniques in many areas of medicine. This trend might also increase the attractiveness for the clinical implantation of this valuable diagnostic tool in in- and outpatient clinic routines. 

## 5. Conclusions

According to the authors‘ point of view, the ultrasonographic indirect measurement of humeral torsional side difference is an easy, valid, and radiation-free method that can be used for patients with healed proximal humerus fractures and humeral shaft fractures. 

## Figures and Tables

**Table 1:** Case report: Passive range of rotational motion in 90° of shoulder abduction.

Case Study: Passive Range of Rotational Motion in 90° Abduction		
	Internal rotation (in degrees [°])	External rotation (in degrees [°])
Left arm (unaffected)	65°	100°
Right arm (affected)	0°	140°

**Table 2:** Different sonographic torsion measurements in previous literature.

Different Sonographic Torsion Measurements in Previous Literature				
Author	Year	n	Torsion Angle (in degrees [°]) Mean ± Standard Deviation (Range)	Journal
Determining the torsion angle using the forearm and measuring the angles in relation to a vertical reference axis				
Ito et al. [6]	1995	30	15.1 ± 3.9 (right) ± 2.9 (left)	J. Shoulder Elbow Surg.
Whiteley et al. [11]	2006	32	18.2 ± 9.6 (4 to 40) (dominant) 19.8 ± 10.8 (5 to 45) (nondominant)	J. Sci. Med. Sport
Whiteley et al. [11]	2006	70	13.8 ± 8.6 (−3 to 36) (dominant) 25 ± 9.2 (8 to 50) (nondominant)	J. Sci. Med. Sport
Thomas et al. [14]	2012	48	0.53 ± 12.53 (dominant) 16.13 ± 11.53 (nondominant)	J. Shoulder Elbow Surg.
Shanley et al. [15]	2012	66	10 ± 11 (dominant) 23 ± 11 (nondominant)	J. Shoulder Elbow Surg.
Noonan et al. [16]	2015	324	10.4 ± 11.7 (dominant) 22.7 ± 11.7 (nondominant)	Am. J. Sports Med.
Achenbach et al. [12]	2019	276	16.5 ± 9.6 (dominant) 13.5 ± 9.4 (nondominant)	Knee Surg. Sports Traumatol. Arthrosc.
Yaari et al. [13]	2020	40	20 ± 10 (dominant) 29 ± 12 (nondominant)	Int. J. Sports Phys. Ther.
Determining the torsion angle using the forearm and measuring the angles in relation to a horizontal reference axis				
Myers et al. [8]	2012	24	74.2 ± 14.5 (dominant) 61.2 ± 14.4 (nondominant)	Am. J. Sports Med.
Saka et al. [9]	2015	28	78.5 ± 7.9 (dominant) 70.1 ± 7.8 (nondominant)	Orthop. J. Sports Med.
Hannah et al. [10]	2018	30	64.4 ± 9.5	J. Athl. Train.
Sonographic measurement of retroversion according to Harland				
Jerosch et al. [37]	1989	20	67.85 ± 7.1 (52 to 80)	Ultraschall Med.
Harland et al. [4]	1991	20	70.65 ± 8.4 (52 to 86)	Z. Orthop.
Harland et al. [4]	1991	111	60.9	Z. Orthop.
Kunz et al. [34]	1993	40	52.0 ± 13.1 (24 to 75)	Z. Orthop.

## Data Availability

Data are available on request.
